# Cerebrovascular Tetraptych

**DOI:** 10.1161/SVIN.125.002124

**Published:** 2026-01-06

**Authors:** Emma Hall, Björn M. Hansen

**Affiliations:** Department of Radiology, Skåne University Hospital, Lund, Sweden (E.H., B.M.H.).; Department of Clinical Sciences, Stroke Imaging Research Group, Lund University, Sweden (E.H., B.M.H.).

## About the Artist

Emma Hall, MD, is a radiology resident, and Björn Hansen, MD, PhD, is a fellow in neurointerventional radiology. Both are clinically active at Skåne University Hospital in Lund and conduct research within the Stroke Imaging Research Group at Lund University. Their research focuses on clinical outcomes after endovascular thrombectomy for ischemic stroke and the development of novel imaging techniques for patients with stroke.

## About the Technique

The images are derived from a single digital subtraction angiography image of a unilateral internal carotid artery contrast injection, which was mirrored, refined, and digitally enhanced for artistic purposes. The vessels were manually enhanced using Adobe Photoshop (Adobe Inc, San Jose, CA), and the images were then colored using Procreate (Savage Interactive Pty Ltd, North Hobart, Australia).

## Artist Perspective

These images illustrate the intricate network of arteries in the anterior circulation of both hemispheres of the brain, inspired by the 1960s and 1970s pop art movement but with a more contemporary color palette.

**Figure:**
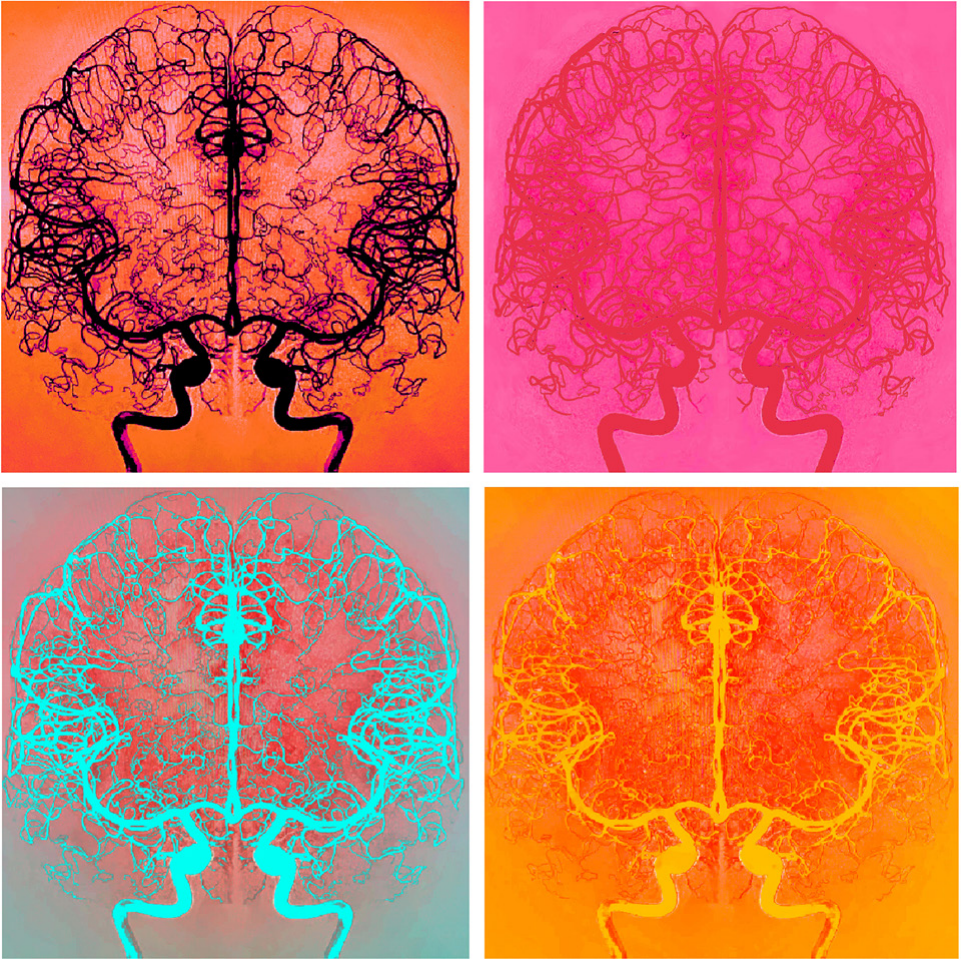


## ARTICLE INFORMATION

### Acknowledgments

The authors thank Johan Wassélius, MD, PhD, for creative input, and all colleagues and patients at the Department of Medical Imaging and Physiology at Skåne University Hospital, Lund.

### Sources of Funding

This work was funded by ALF (E. Hall), Teggerstiftelsen (E. Hall), SUS Stiftelser och Donationer (B.M. Hansen), and Regionala Forskningsmedel (B.M. Hansen).

### Disclosures

This is original artwork created by the author that has not been published, partially or entirely, elsewhere. The authors report no conflicts.

